# Long-term effects of early adversity on the mental health of college students: The mitigating effect of physical exercise

**DOI:** 10.3389/fpsyg.2023.1102508

**Published:** 2023-02-15

**Authors:** Xinzhu Wang, Kaixian Fu

**Affiliations:** ^1^School of Teacher Education, Xichang University, Xichang, Sichuan, China; ^2^Development Planning Division, Xichang University, Xichang, Sichuan, China

**Keywords:** mental health, early adversity, physical exercise, mitigating effect, college student

## Abstract

**Background:**

We aim to investigate the long-term effects of early adversity on university students’ mental health and the mitigating role of physical exercise on this effect.

**Methods:**

The survey sample consisted of 895 college students. Descriptive statistics, linear regression, and moderating effect analysis were used to analyze the results.

**Results:**

(1) Early adversity negatively predict mental health level (*β* = −0.109, *t* = −4.981, *p* < 0.01); (2) Physical exercise can effectively mitigate the long-term harm of early adversity to mental health (*β* = 0.039, *t* = 2.001, *p <* 0.05); compared to low-level physical exercise (*b*_simple_ = −0. 067, *t* = −7.88, *p* < 0.01), high-level physical exercise can mitigate the long-term harm of early adversity to mental health (*b*_simple_ = −0, 025, *t* = −2. 37, *p <* 0.01).

**Conclusion:**

Early adversity affects the mental health of university students, but physical exercise can effectively mitigate this effect.

## 1. Introduction

Nowadays in China mental health of university students has been a difficulty and pain point in the administration of universities. Many factors such as academic pressure, interpersonal conflict, and job-hunting stress and so on were documented to be related with students’ mental health (Luzhu and Huiming, 2022). In recent years, however, some Chinese scholars have begun to take the perspective of life course theory (LCT) to probe deeply into the origin of mental illness for students in universities. For instance, a study demonstrated that family adversities in the first 12 years in life could significantly predict the level of depression for university students (Xun, 2022). Another study found that childhood abuse experience were correlated with suicidal ideation for university students (Zhenyi and Zhanfeng, 2021). A review proposed that early growth adversity was closely related to various mental diseases in the whole life course (Wanxu and Ying, 2021). The present study aimed to examine the relationship between early adversity and mental health for college students from the perspective of LCT, and based on the proactive health theory (PHT) to explore how physical exercise could moderate their relationship.

### 1.1. Life course theory, early adversity and mental health

According to LCT, early adversity refers to the adverse events or trauma experienced by individuals in early life, such as family poverty, severe illness or unexpected bereavement of family members, parental divorce, emotional or physical abuse, peer bullying, etc. (Misiak et al., 2022) In terms of early life, some researchers defined it as the life period before the age of 18, while others defined it as the period from birth to adolescence (Mingxue and Chao, 2017). Just as the study by Xun (2022), the current paper limited the early life in the first 12 years of life course. Hence, in the present paper, early adversity referred to the adverse experiences before the age of 12.

Life course theory proposes that early adversity has long-term effects on individuals’ physical and mental health, resulting in the individual’s physical and mental health disadvantages throughout life (Matthias and Klaus, 2016; Bellis et al., 2019; Sieber et al., 2020). Some studies demonstrated that adolescents who experience early adversity tend to show more behavioral problems (Barker, 2018; Wenming et al., 2020), and early adversity is significantly associated with depression and anxiety in middle school students (Junmei et al., 2020). Compared to university students who grew up living with their parents, those who had been left behind in childhood were reported to have higher levels of mental disorders, including suicidal ideation (Liu et al., 2021). Other investigations proposed that early adversity can put individuals in a physiological and psychological state of latent unhealthiness from childhood to early adolescence (Duffy et al., 2018), and along with various stresses, these individuals often develop into mental illness at clinical level in adulthood (Zhilei and Zhiming, 2018; Zhiyuan et al., 2021). Analyses of representative longitudinal data showed that low parental education levels, childhood poverty, and child emotional neglect can lead to mental illness in middle-aged and older people (Ferraro et al., 2016; Halonen et al., 2017). Similar studies also have corroborated the idea that early adversity significantly predicts the physical and mental health of older people (Yang and Lou, 2016; Li and Lu, 2020).

There are three reasons why early adversity has a long-term impact on individuals’ mental health. First, the stress incurred by early adversity can change the properties of macrophages, making them pro-inflammatory, which creates a microenvironment for the development of chronic diseases (O'Connor et al., 2020; Katja et al., 2022); second, stress caused by adversity can increase individuals’ threat vigilance, reduce trust level and self-regulation ability, and give rise to unhealthy lifestyles and coping strategies (Miller et al., 2011); third, early adversity directly damages the individuals’ cognitive ability and brings about potential emotional disorder (Nweze et al., 2022). To sum up, early adversity leads to poor health in early life, which places individuals at a disadvantage in future competition and makes them experience more adversities at later stages, such as low wages, unemployment, and family stress (Wickrama et al., 2008). These subsequent adversities, in turn, damages physical and mental health, causing issues like anxiety, depression, loss of physical function, and even death (Adda et al., 2003; Yanbi and Jingming, 2019).

### 1.2. Proactive health theory, physical exercise and mental health

Proactive health theory (PHT) provides an insight into dealing with the long-term harm of early adversity on mental health. PHT is a newly emerged Chinese local health management theory (Xuxi et al., 2019; Xiangchen and Mengsun, 2020; Chuansheng, 2021). PHT holds that (1) everyone is the first responsible person for his own health. Only by giving play to his subjective initiative and taking the initiative to pursue health, can he maintain physical and mental health; (2) Prevention is more important than treatment. In the latently unhealthy stage, one should actively adjust himself to prevent the development from latently unhealthiness to illness at clinical level; (3) Body and mind are interrelated with each other. Controllable and active stimulation to the body can promote mental health, and the vice versa; (4) A healthy lifestyle is very important for health, including sleep, diet, exercise and psychological adjustment; (5) Physical exercise is a good medicine. It can not only improve physical health, but also enhance mental health and prevent mental illness.

PHT attaches great importance to physical exercise, and believes that physical exercise is an effective way to promote mental health. Empirical research has documented that physical health can boost mental health in differing life stages. A longitudinal study showed that children’s participation in sports could not only improve their mental health in childhood, but also predict their mental health in late adolescence (Li et al., 2021). Surveys of adolescents have shown that physical exercise can improve mental health directly, and it can also achieve this indirectly by improving mental resilience and reducing the perception of academic stress (Hu and Lingling, 2022). Surveys conducted during the COVID-19 pandemic have illustrated that appropriate physical exercise during quarantine can significantly ease anxiety and depression for adults (Dong-dong and Yin, 2021). Previous studies on elderly health have displayed that physical exercise has a very significant effect on promoting mental health (Hekmati Pour and Hojjati, 2016; de Oliveira et al., 2019; Callow et al., 2020). Moderate-and high-intensity exercise can significantly improve the mental health of young seniors (Fangfang and Jiao, 2021). Furthermore, a review found that exercise intervention significantly facilitates the recovery of mental illness, including depression, schizophrenia, bipolar disorder or anxiety disorder, etc. (Bottaccioli et al., 2021). In line with PHT and empirical research, the Chinese government has strongly encouraged citizens to take the initiative to participate in physical exercise (Huarong, 2022), and more and more citizens begin to actively participate in exercise activities nowadays in China (Yang and Youbing, 2021).

There is a complex mechanism for physical exercise to improve mental health. Modern research points out two routes. One is the direct psychological route, that is, physical exercise can lead to positive cognition and positive emotions, as well as the improvement of social interaction (Yan-Lin et al., 2014); the second is the indirect physiological path, that is, physical exercise can facilitate positive physiological changes, such as better heart rate variability and the secretion of neurotransmitters, etc., thus affects mental health (Wei and Zhixiong, 1995). Traditional Chinese medicine theory believes that there are seven emotions and five spirits for a human being, and they are linked to different organs; and physical activity can relax the whole body and improve the function of organs, through which it adjusts the corresponding “seven emotions and five spirits” so that the mental state eventually becomes better (Yumin and Wenlong, 1996).

### 1.3. Hypotheses

In China, many students in universities have experienced multiple childhood adversity that have produced potential long-term harm to their mental health (Li et al., 2021). According to PHT, one is responsible for his health to a large extent, if one takes initiatives to pursue health, he can maintain health in body and mind, and physical exercise can be beneficial to mental health through active stimuli to body. From this logic, it can be inferred that those who take proactive and healthy behaviors can overcome the long-term harm on their mental health caused by early adversity to a certain extent. However, there is no empirical research to show that physical exercise for university students can effectively mitigate the long-term effects of early adversity on their mental health. Aiming to fill this gap and provide empirical support for PHT in terms of mental health, this study investigated the impact of early adversity on the mental health of university students with a focus on the mitigation effect of physical exercise.

Based on the above-mentioned basis, the current study proposed two hypotheses:

1. For university students, early adversity negatively predicts their mental health.
2. Physical exercise can effectively mitigate the long-term harm of early adversity to their mental health.

## 2. Materials and methods

### 2.1. Participants

The participants in this study were students from five “second-tier” universities in three provinces in China (Guangxi, Yunnan, and Guizhou). Through an online survey platform, in total 895 questionnaires were collected from the participants, of which 427 (47.81%) were males while 468 (52.19%) were females; in terms of the identity, 193 (21.60%) participants were class leaders while 702 (78.40%) were ordinary students; in terms of identy, 827 (92.43%) participants were Han and 68 (7.57%) were from ethnic minority groups; in terms of religious belief, 101 (11.30%) participants practiced religion while 794 (88.70%) did not; in terms of the origin of students, 581 (64.94%) participants were from rural areas while 314 (35.06%) were from urban areas. The mean age of the participants was 22.7 (SD = 3.64; range = 18–25)and the mean height was 164.22 cm (SD = 3.55).

### 2.2. Methods and procedures

Early adversity in this study was measured using the Childhood Environmental Conditions Scale created by Ellis et al. (2009). The scale includes 14 items, with each item scored on a 5-point Likert scale, ranging from “1 = never” to “5 = often.” Participants were asked to recall their experiences of early adversity before the age of 12, including starvation, physical and mental abuse, physical and mental neglect, sexual assault, etc. The mean value of all items was the early adversity level. The higher the mean, the more unfavorable the childhood environment was. In this study, the Cronbach’s coefficient of the scale was 0.79.

Mental health was measured using the WHO-5 well-being index. This scale has been commonly used in China and has good reliability and validity (Heun et al., 2001; Liu Xingdi et al., 2014). It consists of five items and requires the subjects to report their mental states (e.g., moods and sleeping) in the last 2 weeks. This measurement also adopted a 5-point Likert scale, ranging from “1 = never” to “5 = always.” The higher the mean, the better the mental health. In this measurement, the Cronbach’s coefficient of the five items was 0.75, showing good reliability.

Following the method reported by Andersen et al. (2022), physical exercise was measured by asking participants to report the frequency of active physical activity (such as brisk walking, jogging, swimming, ball games, etc., which lasts for more than 30 min each time) each week during the past year, a 5-point Likert scale was used. Integers from 1 to 5 represented “once a week or less,” “twice a week,” “three times a week,” “four times a week,” and “five times a week or more.”

In addition, we asked the participants about their height in the questionnaire. Height is an effective proxy variable of early health (Gao and Smyth, 2010). Taking the height and origin of students as control variables can effectively remove the confounding early factors in the data analysis, so that we can better investigate the mitigating effect of physical exercise.

### 2.3. Tools of data analysis

In the present study, we first used descriptive statistics and Pearson correlation analysis to present preliminary results, and then we used linear regression analysis to examine the long-term predictive effect of early adversity on mental health, and finally health. Finally, we conducted a moderating effect analysis to examine whether physical exercise can mitigate the long-term harm of early adversity to mental health.

## 3. Results

### 3.1. Descriptive statistic

Descriptive statistics are presented in Table 1. The mean value of mental health was 3.86, well above the median of 3 on the 5-point Likert scale. The mean value of early adversity was 1.73 and the mean value of physical exercise was 2.46. Both were below the median of 3 on the 5-point Likert scale. Pearson correlation analysis showed that there was a significant negative correlation between mental health and early adversity (*r* = −0.163, *p* < 0.01) and a significant positive correlation between mental health and physical exercise (*r* = 0.164, *p* < 0.01). Early adversity and physical exercise were significantly and negatively correlated (*r* = −0.222, *p* < 0.01).

**Table 1:** Descriptive statistics and Pearson correlation analysis of key variables (*N* = 8,953).

	Mean	SE	Mean	Early adversity	Age	Height
Mean health	3.86	0.70	1			
Early adversity	1.73	1.17	−0.163^**^	1		
Age	22.73	3.64	−0.013	0.051	1	
Height	164.22	3.55	0.109^**^	−0.117^**^	−0.006	1
Physical Exercise	2.46	1.52	0.164^**^	−0.222^**^	0055^**^	0.101^**^

^*^ indicates that *p* < 0.05, ^**^ indicates that *p* < 0.01, same hereinafter.

### 3.2. Mean values of early adversity and mental health

Table 2 presents the mental health level of university students with different mean values of early adversities.

**Table 2:** Mean values of early adversity and mental health.

Mean values of early adversity	*N*	Percentage	Cumulative percentage	Mental health	
				*M*	SD
1	241	26.93	26.93	4.04	0.62
2	221	24.69	51.62	3.92	0.68
3	189	21.12	72.74	3.82	0.71
4	140	15.64	88.38	3.72	0.73
5	104	11.62	100	3.69	0.71
Total	895	100		3.86	0.7

Table 2 demonstrates that for 15.64% of university students, the mean value of early adversity was 4, and for 11.62% of university students, the mean value of early adversity was 5. This indicated that in underdeveloped Chinese provinces like Guangxi, Guizhou, and Yunnan, a considerable proportion of university students experienced many adversities in their early life.

Table 2 also demonstrates that the higher level of early adversity the university students reported, the lower their level of mental health. For example, university students with a mean adversity value of 1 had a mean mental health value of 4.04; university students with a mean adversity value of 3 had a mean mental health value of 3.82, and when the mean value of early adversity was 5, the mean mental health value was 3.69. This suggests that early adversity had a cumulative long-term effect on mental health (Zhilei and Zhiming, 2018).

### 3.3. Regression analysis and moderation analysis

The results of linear regression analysis are presented in Table 3. To clearly show the predictive effect of early adversity and physical exercise, regression 1 was regarded as the baseline level, and only several control variables-age, height, gender, the origin of students, identity at school, and religious belief -were included. Regression 1 was quite significant (*F* = 60.61, *p* < 0.01, *R*^2^ = 0.045). The positive predictive effect of height on mental health was significant (*β* = 0.063, *t* = 5.712, *p* < 0.01), indicating that higher students were more physically healthy; the positive predictive effect of the origin of students on mental health was significant (*β* = 0.180, *t* = 14.512, *p* < 0.01), which suggested that the mental health level of urban students was higher than that of rural students; identity at school had a significant positive predictive effect on mental health (*β* = 0.060, *t* = 5.763, *p* < 0.01), which suggested that mental health of class cadres is better than that of ordinary students. Neither gender nor religious belief had significant predictive effects.

**Table 3:** Linear regression analyses of mental health.

	Regression 1		Regression 2		Regression 3	
	*β*	*t*	*β*	*t*	*β*	*t*
(Intercept)		60.555^**^		59.420^**^		56.489^**^
Age	−0.001	−0.122	0.007	0.592	0.008	0.638
Height	0.063	5.712^**^	0.057	5.262^**^	0.057	5.205^**^
Gender	0.004	0.405	−0.002	−0.142	−0.002	−0.147
Origin of students	0.180	14.512^**^	0.106	7.593^**^	0.106	7.61^**^
Ethnic group	0.011	1.456	0.012	1.073	0.012	1.082
Religious belief	−0.007	−0.648	−0.004	−0.41	−0.004	−0.408
Identity at school	0.060	5.763^**^	0.067	6.472^**^	0.066	6.345^**^
Early adversity			−0.08	−6.090^**^	−0.109	−4.981^**^
Physical exercise			0.111	10.100^**^	0.084	4.309^**^
Physical exercise * Early adversity					0.039	2.001^*^
*F*	60.61^**^		64.95^**^		58.76^**^	
*R* ^2^	0.045		0.061		0.062	

Baseline groups were female, rural students, students from the ethnic minority group, students without religious belief, and ordinary students, respectively.

Regression 2 included early adversity and physical exercise. Compared to regression 1, the explanatory power of regression 2 was enhanced (*F* = 64.95, *p* < 0.01, *R*^2^ = 0.061). The negative predictive effect of early adversity on mental health was significant (*β* = −0.08, *t* = −6.090, *p <* 0.01), and the positive predictive effect of physical exercise on mental health was also significant (*β* = 0.111, *t* = 10.100, *p* < 0.01).

Regression 3 integrated the early adversity*physical exercise interaction. However, results showed that the explanatory power of the model was not significantly raised (*F* = 58.76，*p* < 0.01, *R*^2^ = 0.062). There was a significant negative predictive effect of early adversity on mental health (*β* = −0.109, *t* = −4.981, *p* < 0.01) and a significant positive predictive effect of physical exercise on mental health (*β* = 0.084, *t* = 4.309, *p* < 0.01). The predictive effect of early adversity*physical exercise interaction on mental health was positive and reached the significant level (*β* = 0.039, *t* = 2.001, *p* < 0.05). Taken together, it is suggested that physical exercise can significantly moderate the long-term effect of early adversity on mental health.

**Figure 1:**
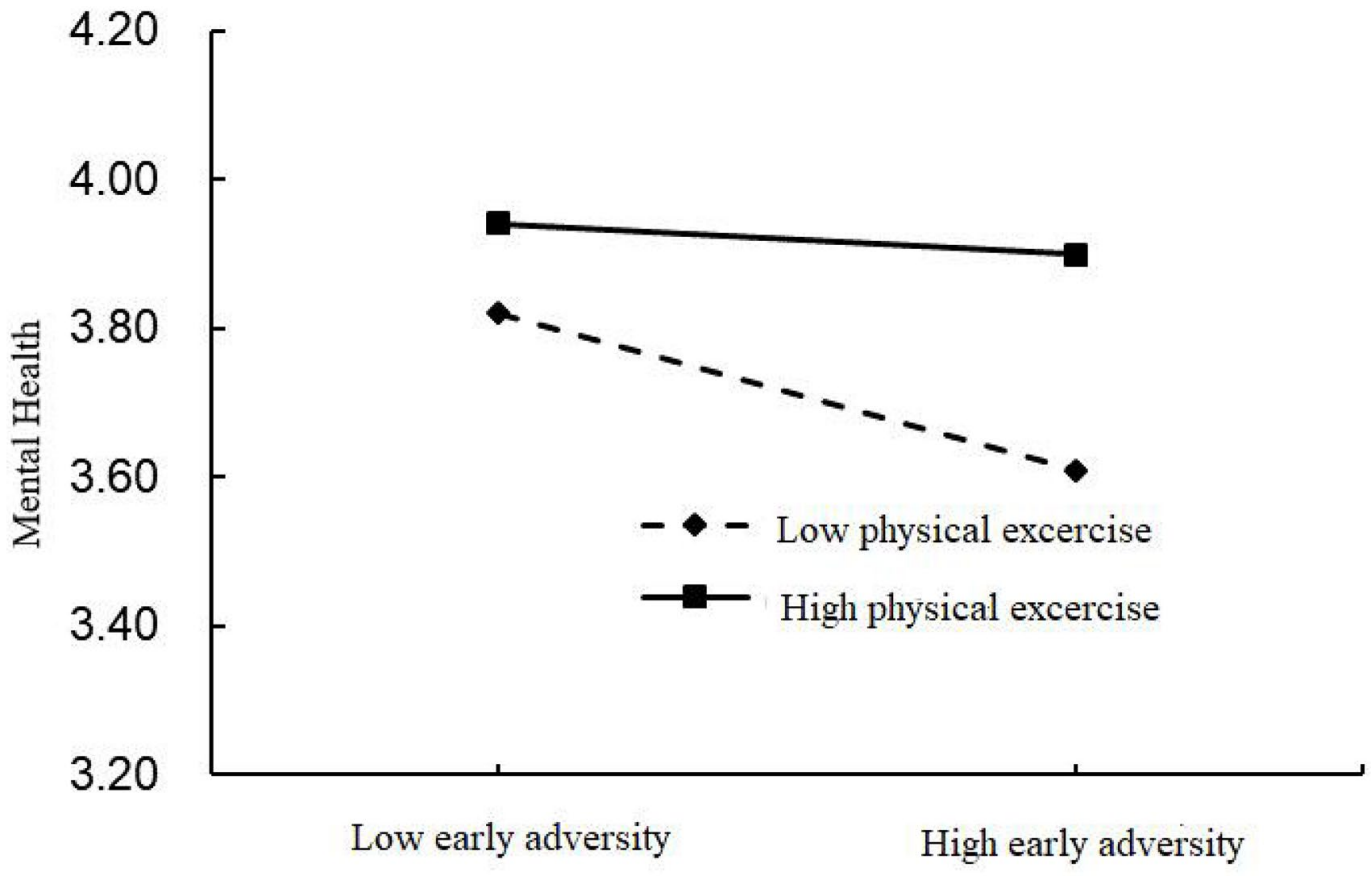
Moderating effects of physical excercise.

To present the moderating effect of physical exercise more straightforwardly, using the research of Dearing and Hamilton (2010) as a reference, a simple slopes graph was drawn below (see Figure 1).

Simple slopes analysis illustrates that when the value of physical exercise was 1 standard deviation smaller than the mean, early adversity could significantly and negatively predict mental health (*b*_simple_ = −0. 067, *t* = −7.88, *p* < 0.01); When the value was 1 standard deviation above the mean, early adversity could also significantly and negatively predict mental health (*b*_simple_ = −0. 025, *t* = −2.37, *p* < 0.05), however, its predictive power was reduced. Such results indicate that the long-term effects of early adversity on mental health are not easy to remove and do not completely disappear even with higher levels of physical exercise; on the other hand, however, physical exercise does mitigate such long-term harm. The marginal effect of the long-term effect of early adversity was 0.067, and its upper and lower bounds were [−0.0896, −0.0356], while at high physical exercise level this marginal effect was decreased to 0.025, with the upper and lower bounds being [−0.0375, −0.0192] although it remained significant.

## 4. Analysis and discussion

### 4.1. The long-term effect of early adversity

The present study examined the relationship between early adversity and mental health in university students. Regression analysis revealed that, after controlling the variables of age, height, gender, the origin of students, identity at school, and religious belief, early adversity could significantly and negatively predict mental health, so Hypothesis 1 was fully verified (shown in regression 2 in Table 3). This result also corroborated the previous research findings. For example, a longitudinal analysis of health and retirement in China showed that early life family environment was significantly associated with depression in old age (Chen and Tan, 2021). Another longitudinal study also pointed out that family adversity in childhood could directly or indirectly impair physical and mental health in adulthood (Wickrama et al., 2005). Some studies have suggested that the stress caused by early adversity gave rise to a physiological imprinting effect, i.e., the liability to inflammation at the cellular level. On the one hand, this effect creates the microenvironment of physiological diseases; on the other hand, individuals who suffer from this effect may become over-sensitive to stressful events. The imprinted effect leaves lifetime harm to individuals (Gluckman et al., 2008). Chinese researchers have used a two-fold cumulative effect to explain this kind of imprinted harm: the more types of early adversity and the longer the time, the more significant the physical and mental development disadvantages of the individual (see Table 2). The physical and mental development disadvantages give rise to many adversities in competition in adulthood, which leads to low levels of physical and mental health as a result (Zhilei and Zhiming, 2018). Finally, data analyses also indicated that the height of university students could significantly predict their mental health, suggesting that early health, which was deeply influenced by early adversity, was closely related to mental health level in adulthood (Yanbi and Jingming, 2019).

### 4.2. The mitigating effect of physical exercise

According to the PHT, this study proposed hypothesis 2, which stated that physical exercise could effectively mitigate the long-term effect of early adversity on mental health. Moderating effect analysis suggested that the early adversity*physical exercise interaction could significantly predict mental health. A follow-up simple slopes analysis found that long-term harm incurred by early adversity on mental health remained significant regardless of the exercise level. However, compared to low-level physical exercise (*b*_simple_ = −0. 06), high-level physical exercise could reduce the predictive effect of early adversity on mental health to a certain extent (*b*_simple_ = −0.025). This showed that physical exercise could effectively mitigate the long-term damage to mental health caused by early adversity. Thus, hypothesis 2 was also supported. Previous studies have shown that physical exercise not only enhances mental health through psychological effects directly (such as inducing positive thinking and emotions, as well as increasing social support), but also indirectly, through physiological effects (such as improving heart rate variability, stimulating the secretion of neurotransmitters; Wei and Zhixiong, 1995). Traditional Chinese medicine uses the theory of viscera to explain the abovementioned effects, arguing that physical exercise can boost the functionality of the organs and regulate spirits, so as to improve the mental state.

Moderation effects analysis also suggested that the long-term harm of early adversity on mental health was intractable and could not be entirely removed by physical exercise in adulthood. The main reason was that early adversity makes an individual have a health disadvantage before adulthood, which not only makes the individual more sensitive to stress in adulthood but also brings about more adversities (such as unemployment, low income, physical illness, etc.) to the individual because of the disadvantages in competition during adulthood (Yanbi and Jingming, 2019). In summary, early adversity predisposes individuals to poorer health and leads to disadvantages in competition during adulthood. That is the reason why physical exercise in adulthood is not adequate to completely compensate the mental health damage from early adversity.

## 5. Implications

The present study explored the relationship between early adversity and mental health of college students. Regression analysis showed that early adversity has long-term effects on college students’ mental health; meanwhile, moderating analysis showed that proactive health behavior (such as physical exercise) can significantly mitigate these long-term effects. In this way, the two hypotheses based on life course theory and PHT were all well supported. The current investigation has its theoretical and practical implications.

### 5.1. Theoretical implications

Theoretically, this study supported the viewpoint in LCT that early adversity can produce long-term harm to mental health of individuals, and the harm is profound and stubborn. This deepened the current understanding in the origins of mental problems. Traditionally, scholars in China have a mindset that mental health for adults is largely determined by current stress factors, while ignoring the basic role of early experience. This research helps to change this academic mindset and inspire the research in LCT in China.

On the other hand, this study supported the view of PHT, that is, proactive health behaviors (such as physical exercise) can enhance mental health effectively, and mitigate the long-term effects of early adversity on mental health, although it cannot completely cure these harmful effects. According to the PHT, mental health can be enhanced by actively stimulating the body through physical exercise, which shows that from the perspective of mental health body and mind are closely linked with each other. In addition, the PHT is a newly emerged health management theory in Chin, which remains to be theoretical speculation at present. The present empirical investigation is conducive to the development of this theory.

### 5.2. Practical implications

Practically, the mental health issue of university students is a major concern of Chinese society (Wang et al., 2019). In some second-tier colleges and universities in underdeveloped areas, a considerable number of students have experienced many early adversities. They have low levels of mental health and are prone to developing significant mental illness under stress. In order to effectively improve the mental health of students, universities should promote proactive health behaviors in the administration and encourage students to actively do physical exercise, especially for those who have experienced more early adversities (e.g., former left-behind children and students from low-income families). Physical exercise is a good medicine; it can effectively mitigate the long-term harm of early misfortune on the mental health of university students, and enhance their internal strength to resist mental pressure, thereby preventing the further development from the state of latently unhealthiness to the state of illness at clinical level.

In addition, mental health is related closely to early adversity. According to the PHT, early prevention and elimination of latent unhealthiness are very important for the mental health of future college students. Therefore, Chinese government and schools at all levels should pay early attention to children’s early life experiences and provide economic and psychological assistance to those children who are experiencing adversities, so as to fundamentally reduce the occurrence of mental diseases in future.

## 6. Limitations

The present study had some limitations. Regarding measuring physical exercise, the measurement in this paper could not capture features like the ways of doing exercise and the intensity of exercise; meanwhile, cross-sectional data can only reveal correlations but not causality, and this study cannot draw a causal relationship between early adversity and mental health; moreover, proactive health behaviors include not only physical exercise but also diet, sleep, and self health adjustment, etc. Examining physical exercise alone cannot fully unveil the significant role of proactive health behaviors in mitigating the long-term effects of early adversity on mental health. Finally, PHT is a newly emerged health theory based on the Chinese culture, and its elaboration on mental health needs to be deepened and completed, which makes the current study weak in theoretical basis. Future studies should try to fill these gaps to better demonstrate that proactive health behavior can effectively mitigate the long-term effects of early adversity on mental health.

## Data availability statement

The original contributions presented in the study are included in the article/Supplementary material, further inquiries can be directed to the corresponding author.

## Author contributions

KF was responsible for data processing. XW was responsible for writing the whole thesis. All authors contributed to the article and approved the submitted version.

## Funding

This work was supported by Key Research Base of Humanities and Social Sciences (Leisure Sports Industry) of Sichuan Provincial Department of Education (XXTYCY2022B15): Early Adversity and Mental Health Inequality: A Study on the Compensation Effect of Leisure Sports; The 2022 Project of Sichuan Education Development Research Center (CJF22035): Research on the Mental Health of Rural School Teachers in Liangshan Yi District under the Double Reduction Policy. The 2022 Annual Project of Sichuan Mental Health Education Research Center (XLJKJY2211B): Long term Injury of Early Adversity and Compensation Effect of Active Health Behavior; School of Teacher Education of Xichang University (LGLS202201): College Teachers’ mental health: Self induced and prevention under pressure.

## Conflict of interest

The authors declare that the research was conducted in the absence of any commercial or financial relationships that could be construed as a potential conflict of interest.

## Publisher’s note

All claims expressed in this article are solely those of the authors and do not necessarily represent those of their affiliated organizations, or those of the publisher, the editors and the reviewers. Any product that may be evaluated in this article, or claim that may be made by its manufacturer, is not guaranteed or endorsed by the publisher.
